# Reduction of CFU-GM and circulating hematopoietic progenitors in a subgroup of children with chronic neutropenia associated with severe infections and delayed recovery

**DOI:** 10.1371/journal.pone.0213782

**Published:** 2019-03-14

**Authors:** Fabio Timeus, Nicoletta Crescenzio, Luiselda Foglia, Alessandra Doria, Maria Giuseppina Stillitano, Emanuela Garelli, Raffaela Mazzone, Laura Vivalda, Stefano Vallero, Ugo Ramenghi, Paola Saracco

**Affiliations:** 1 Pediatric Onco-Hematology, Regina Margherita Children Hospital, Turin, Italy; 2 Pediatric Hematology, Regina Margherita Children Hospital, University of Turin, Turin, Italy; 3 Biochemistry Laboratory, Regina Margherita Children Hospital, Turin, Italy; 2nd medical school of Charles University, CZECH REPUBLIC

## Abstract

Myelopoiesis was evaluated in 66 pediatric patients with chronic neutropenia who were positive for anti-neutrophil antibodies (median age at diagnosis: 11 months, median neutrophil count at diagnosis: 419/μl). Other causes of neutropenia were excluded. Bone marrow morphology, clonogenic tests and/or the peripheral blood CD 34+ cell count, and apoptotic rate were evaluated in 61 patients with neutropenia lasting > 12 months or severe infections. The peripheral blood CD 34+ cell count and apoptotic rate were evaluated in five patients with shorter neutropenia. The median follow-up time was 29 months (range 7–180 months). Forty-seven patients (71.2%) had a spontaneous recovery after 7–180 months (median 29 months). The group of patients younger than 24 months at diagnosis (n = 50) had a higher probability of recovery (40/50 vs. 7/16 χ^2^ p<0.01) with a shorter period of neutropenia (median 26 versus 47 months, Kaplan-Meier analysis p = 0.001). The colony-forming units–granulocyte-macrophage (CFU-GM) were significantly decreased in 26/35 patients (74%) evaluated for clonogenic tests. All patients with normal CFU-GM recovered (9/9 patients); whereas, neutropenia persisted in 12/26 patients with reduced CFU-GM (46%, Pearson χ^2^ p = 0.02). In 36/55 (65%) patients evaluated by flow cytometry we observed reduced circulating CD34+ cells compared with controls of the same age. An increase in the circulating CD34+ cell apoptotic rate was observed in 28/55 patients (51%). Infections requiring hospitalization were observed in 9/18 (50%; Pearson χ^2^, p = 0.03) patients with both decreased circulating CD34+ cells and increased CD34+ apoptotic rates. In the group aged < 24 months, we observed a significant correlation between the persistence of neutropenia and decreased circulating CD34+ cells (Pearson χ^2^ p = 0.008). In conclusion, reduced CFU-GM and circulating hematopoietic progenitors were observed in a subgroup of children with chronic neutropenia who were positive for anti-neutrophil antibodies and had a higher incidence of severe infections and delayed spontaneous remission.

## Introduction

Autoimmune neutropenia of childhood is characterized by low neutrophil absolute counts (in Caucasians <1000/μl up to the age of 1 year, <1500/μl from 1 year to adulthood) due to increased immune-mediated destruction, with a duration that exceeds 6 months. The median age at presentation is 8 to 11 months (range 2–54 months) and spontaneous remission is observed in the majority of patients after a mean of 20 months. The clinical course is benign, with a mild increase in bacterial infections [1–7]. Autoantibodies are directed against neutrophil-specific antigens, mainly FcγRIIIb or CD16b, carrying the human neutrophil antigen polymorphism HNA-1, and less frequently against other targets such as CD11b (HNA-4) [8–13]. Anti-neutrophil antibodies are difficult to detect by standard methods [14, 15, 1] and guidelines [16] suggest repeating the test at least four times if the results are negative. The bone marrow in these patients is usually normal or hypercellular; however, maturation arrest of the neutrophil precursors has been described. [1]

In the past decades, studies using clonogenic assays on a low number of patients have shown a normal number of colony-forming units–granulocyte-macrophage (CFU-GM) in childhood chronic neutropenia. [17, 18]. The aim of this study was to evaluate myelopoiesis in a group of children with isolated chronic neutropenia, who were positive for anti-neutrophil antibodies, using clonogenic tests and flow cytometry for circulating CD34+ cells, as well as to investigate the possible correlations between the morphologic and functional aspects of myelopoiesis and the clinical course.

## Materials and methods

### Patients

In the last 15 years at our center, myelopoiesis was evaluated in 66 pediatric patients with chronic autoimmune neutropenia (median age at diagnosis: 11 months, median neutrophil count at diagnosis: 419/μl), diagnosed according to the following criteria: neutropenia lasting >6 months, positivity for anti-neutrophil antibodies using the flow cytometry granulocyte immunofluorescence test (GIFT [14]), and the exclusion of other causes of neutropenia. Thirty-nine patients were evaluated using bone marrow morphology and clonogenic tests due to the presence of a more prolonged period of neutropenia (persistent neutropenia after a follow-up of at least 12 months) or the presence of moderate-severe infections requiring hospitalization. Sepsis, pneumonia, skin soft tissue abscesses, osteomyelitis, otomastoiditis, and meningitis/encephalitis were defined as severe infections. Circulating CD 34+ cells and their apoptotic rates were evaluated by flow cytometry in 28 of these 39 patients and in a further 22 patients with persistent neutropenia after 12 months of follow-up or with severe infections. Flow cytometry evaluation of peripheral blood hematopoietic progenitors was performed in five additional patients with a shorter course of neutropenia. The study was approved by the Local Ethical Committee as an observational study performed during diagnostic follow-up (prot n. 24316/C28.1; website: www.cittadellasalute.to.it). Written informed consent was obtained from parents or legal guardians and was stored in the patient’s clinical files.

### Bone marrow morphology

In 39 patients, the bone marrow was evaluated by an expert after May-Grunwald Giemsa staining. More than 1000 nucleated cells were evaluated for each sample. Myeloid, erythroid, and megakaryocyte series were analyzed and evaluated for possible signs of dysplasia and myeloid maturation arrest.

### Anti-neutrophil antibodies

The presence of immunoglobulin G and/or M-type anti-neutrophil antibodies was examined by the indirect GIFT method [14]. A mixture of neutrophils from normal subjects, obtained after red blood cell lysis, was incubated at room temperature with the patient’s serum for 30 min, washed twice and incubated for 15 min with FITC-conjugated polyclonal anti-human IgG and IgM rabbit F(ab’)_2_ antibody-fragments (Dako, Glostrup, Denmark). Binding of antibodies to the neutrophils was analyzed by a FACSCanto (BD Biosciences, San Jose, CA). The neutrophil population, identified by its physical parameters, was defined as positive when >30% of the neutrophils were positive and/or the mean fluorescence intensity was increased by >30% in comparison with a sample treated with control serum from a healthy subject. Samples from children without neutropenia were used as controls.

### Clonogenic assays

For 35 patients, 1 x 10^5^/ml low-density mononuclear cells were obtained from the bone marrow by density centrifugation over a Ficoll-Hypaque gradient. The cells were plated in multiwell plates in Iscove’s modified Dulbecco’s medium (Sigma Aldrich, St. Louis MO, USA) containing 30% fetal calf serum (Sigma Aldrich), 0.3% noble agar, 10 ng/ml rhIL-3 (Invitrogen CA, USA), and 20 ng/ml granulocyte-macrophage colony-stimulating factor (rhGM-CSF, Invitrogen) or 10 ng/ml granulocyte colony-stimulating factor (rhG-CSF). After 14 days, single aggregates of more than 40 cells were scored as CFU-GM or colony forming units–granulocyte (CFU-G). The results were compared with the historical pediatric controls of our laboratory (healthy bone marrow donors), cultured under the same conditions.

### CD34+ cell absolute count and apoptotic index

Flow cytometry analysis was performed within 2 h after venipuncture. The absolute CD34+ cell count and apoptotic rate were evaluated using three-color fluorescence for CD45, CD34, and Annexin V as follows: 5 x 10^5^ nucleated cells were incubated for 20 min at 4°C with anti-CD34 PE (8G12, Becton Dickinson, San José, CA, USA) and anti-CD45 PerCP (2D1, BD). After incubation and erythrocyte lysis using ammonium chloride, the samples were washed in cold Dulbecco’s Phosphate Buffered Saline and incubated with Annexin V FITC (Apoptosis Detection Kit, R&D Systems, Minneapolis, MN, USA), according to the manufacturer's instructions. The cells were then analyzed in a FACSCanto cytometer equipped with an argon laser. CD34+ cells were identified by a sequential gating strategy according to the ISHAGE protocol [19]. Absolute CD34 counts were assessed by a two-platform method with a Sysmex K 4500 Counter (Sysmex Corporation, Kobe, Japan). At least 100 CD34+ cells were evaluated in each experiment. The results were compared with the historical controls of our laboratory [20]. Absolute CD34+ cell counts below the mean-1SD of controls were defined as reduced. The CD34+ cell apoptotic rates higher than the mean+1SD of controls were defined as increased.

### Statistics

The Student’s t-test was used to compare CFUs in patients and controls. Pearson's Chi Square was used to test differences in the recovery rate or infection rate between groups. Survival analyses were performed with the Kaplan-Meier method and were used to describe recovery from neutropenia in different groups of patients. Log-rank statistics were used to test between groups. Calculations and graphs were created using Gnumeric software in Linux.

## Results

The median age and the median neutrophil count at diagnosis of the 66 patients evaluated in the present study were 11 months (range 3–192 months) and 419/μl (10–990) respectively; the median follow-up was 29 months (range 7–180 months). Forty-seven patients (71.2%) had a spontaneous recovery after 7–180 months (median 29 months). The patients aged <24 months at diagnosis (n = 50) had a higher probability of recovery and a shorter period of neutropenia than older patients (recovery in 40/50 vs. 7/16, χ^2^, p<0.01; neutropenia for a median 26 months vs. 47 months, Kaplan-Meier analysis, p = 0.001). Twenty-three of the 66 (37%) patients with a median age of 11 months (range 5–180) had infections requiring systemic antibiotics and hospitalization. Most of them were severe infections (sepsis, pneumonia, skin soft tissue abscesses, osteomyelitis, otomastoiditis, meningitis/encephalitis). Fifteen patients were treated with G-CSF (5–10 μg/kg/day). Three of these 15 patients were treated continuously with G-CSF for 60, 26, and 14 months because of recurrent infections. One patient, never treated with G-CSF, developed refractory cytopenia without cytogenetic alterations 11 years after the diagnosis of autoimmune neutropenia and underwent a successful allogeneic stem cell transplantation.

The bone marrow morphology was evaluated in 39 patients. Maturation arrest was present in six patients and mild myeloid dysplastic features were observed at diagnosis in one patient (different from the patient who developed refractory cytopenia), without clonal transformation after a follow-up of 90 months. The clonogenic tests on bone marrow mononuclear cells were evaluated in 35 of the 39 patients. When we compared the CFU number with our historical controls, the CFU-GM and CFU-G were significantly decreased in 26/35 (74%) and 25/35 (71%) patients, respectively (Table 1).

**Table 1:** Clonogenic assays.

**CFU-GM**	**Decreased (n = 26)**	**Normal (n = 9)**	**Controls**
**mean±SD**	**12±11.2**	**74±43.9**	**74±33.0**
**median (range)**	**7 (0–32)**	**58 (34–153)**	**72 (34–130)**
**CFU-G**	**Decreased (n = 25)**	**Normal (n = 10)**	**Controls**
**mean±SD**	**7.0±9.0**	**63±27.8**	**71±28.0**
**median (range)**	**3 (0–28)**	**59 (34–130)**	**68 (34–140)**

CFU-GM and CFU-G/10^5^ mononuclear cells in 35 neutropenic patients with decreased or normal colonies and in controls. The cut-off for normal values of CFU-GM or CFU-G was 33/10^5^ mononuclear cells.

**Fig 1:**
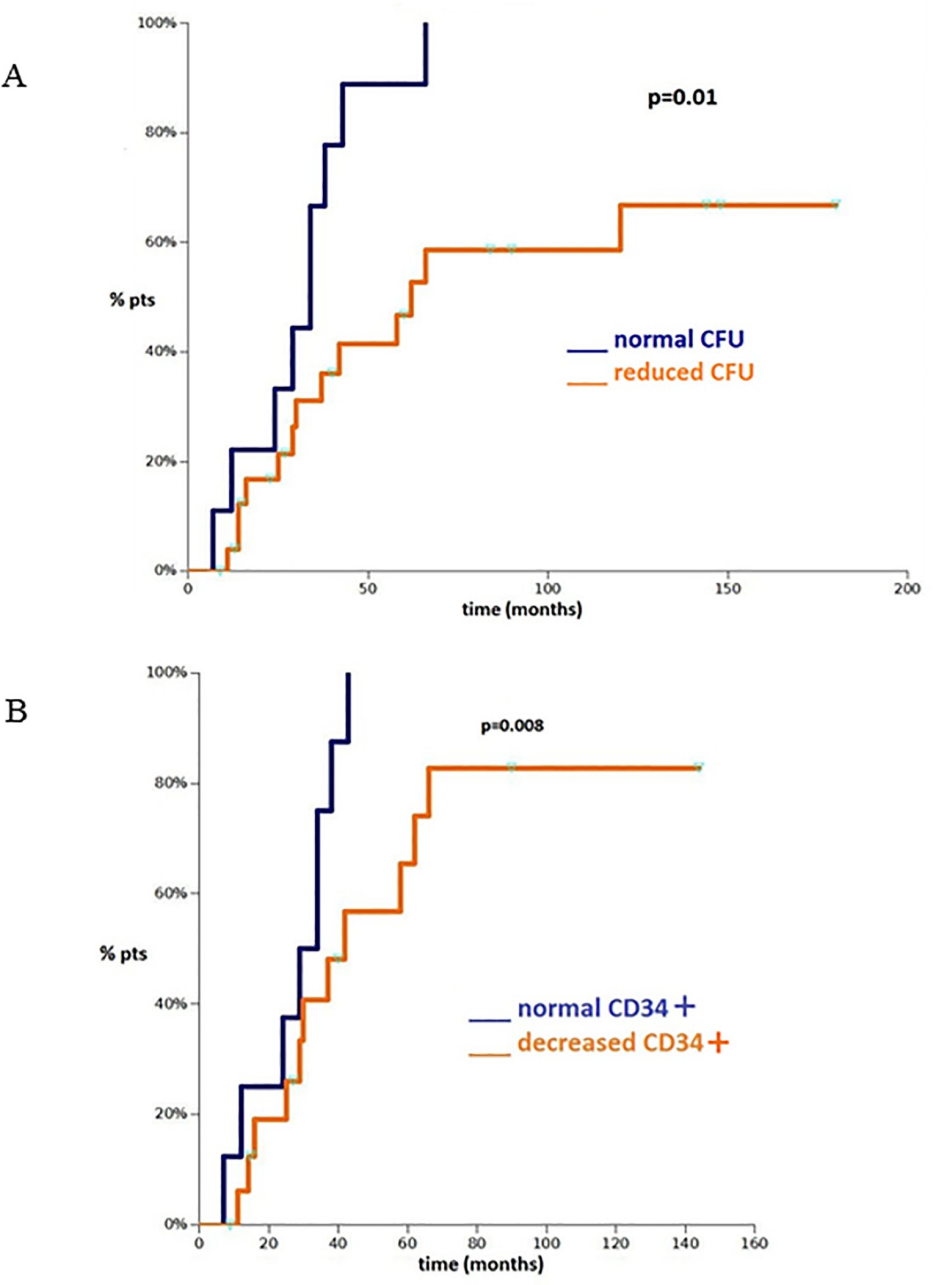
Kaplan-Meier recovery curve. (A) Kaplan-Meier recovery curve for neutropenia for the normal and reduced CFU-GM groups in 35 neutropenic patients (aged 3–192 months) evaluated with clonogenic assays. (B) Kaplan-Meier recovery curve for neutropenia for the normal and decreased circulating CD34+ cell groups of patients aged <24 months (n = 36).

All the patients with normal CFU-GM recovered (9/9 patients), whereas neutropenia persisted in 12/26 patients with decreased CFU-GM (46%, Pearson χ^2^ p = 0.02). There was a similar correlation, although not statistically significant, between the CFU-GM growth pattern and recovery from neutropenia when analyzing the group aged <24 months (Pearson χ^2^ p = 0.06). The results were confirmed by Kaplan-Meier analysis (see Fig 1A). The administration of G-CSF increased neutrophils in 15/15 (100%) patients, and also in those with a reduction of colonies in clonogenic assays.

The circulating CD34+ cells were evaluated in 55 patients. In 36 of the 55 (65%) patients, we observed a reduction in the absolute count of circulating CD34+ cells compared with the controls of the same age [20]. An increase in the circulating CD34+ cell apoptotic rates in 28/55 patients (51%) (Table 2) was also observed. In the other 27/55 patients, the apoptotic rates were unexpectedly below the values of the controls of the same age. Eighteen of the 28 patients with increased CD34+ apoptotic rates also had decreased circulating CD34+ cells.

**Table 2:** Flow cytometric evaluation of peripheral blood hematopoietic progenitors.

Age	CD34+/μl			%AnnexV+		
	Decreased	Normal	Controls	Increased	Normal	Controls
**6–23 months**	**n = 16**	**n = 12**		**n = 14**	**n = 14**	
**mean±SD**	2.1±1.1	6.3±2.7	8.3±4.3	39.6±11.3	9.5±5.1	18.7±10.3
**2–5 years**	**n = 8**	**n = 3**		**n = 5**	**n = 6**	
**mean±SD**	1.63±0.5	4.23±2.4	5.9±3.1	39.02±7.8	12.68±3.4	20.0±2.7
**6–11 years**	**n = 5**	**n = 2**		**n = 3**	**n = 4**	
**mean±SD**	0.95±0.48	3.76±0.06	4.7±1.8	43.13±6.8	9.83±3.6	22.9±3.7
**12–15 years**	**n = 7**	**n = 2**		**n = 6**	**n = 3**	
**mean±SD**	1.21±0.58	2.91±0.57	2.3±1.1	50.4± 11.5	9.87±4.28	27.1±4.8

Peripheral blood absolute CD34+ cell count and apoptotic rate in 55 neutropenic patients and in controls of the same age. Decreased: patients with the absolute count decreased in comparison with the controls of the same age. Increased: patients with the apoptotic rate increased in comparison with the controls of the same age. Normal: patients with normal values.

Moderate to severe infections requiring hospitalization were observed in 13/36 (36%) patients with reduced CD34+ circulating cells versus 4/19 (21%) with normal CD34+ circulating cells and in 12/28 (43%) patients with an increased CD34+ apoptotic rate versus 5/27 (19%) with a normal CD34+ apoptotic rate. When we considered the patients with both decreased circulating CD34+ cells and increased CD34+ apoptotic rates, moderate to severe infections requiring hospitalization were observed in 9/18 (50%; Pearson χ^2^ p = 0.03).

We observed a trend, though not significant, towards the persistence of neutropenia in patients with decreased circulating CD34+ cells (Pearson χ^2^ p = 0.07). This correlation was significant in the group aged <24 months (Pearson χ^2^ p = 0.008). These results were confirmed by Kaplan Meier analysis (Fig 1B). A linear correlation between CFU and circulating CD34+ cells was not statistically significant, perhaps because the majority of flow cytometric evaluations were performed in the group aged <24 months; whereas, the clonogenic tests are more represented in the group aged >24 months.

## Discussion

Autoimmune neutropenia of childhood is a benign disease characterized by spontaneous remission and a low incidence of severe infections. The guidelines for the management of chronic autoimmune neutropenia of childhood [21, 22] suggest avoiding bone marrow evaluation. This is because most patients generally have a benign course of the disease with an unaltered or slightly altered bone marrow morphology. Furthermore, some authors reported normal CFU-GM in autoimmune neutropenia of infancy [17,2,18]. The present study demonstrates a reduction of CFU-GM in a substantial percentage of patients with chronic childhood neutropenia who were positive for anti-neutrophil antibodies and had neutropenia lasting more than one year or the presence of severe infections. The prolonged follow-up excluded the presence of a more complex autoimmune disease and only one case evolved to refractory cytopenia after an 11 year observation period. When we evaluated the peripheral blood hematopoietic progenitors by flow cytometry in a larger group of patients, we frequently observed impaired myelopoiesis. Our findings may have a potential clinical impact: reduced CFU-GM levels were significantly correlated with the duration of neutropenia and moderate-severe infections requiring hospitalization were more frequent in patients with decreased circulating CD34+ cells and increased apoptotic rates. Furthermore, there was a significant correlation between reduced levels of peripheral CD34+ cells and delayed neutrophil recovery in the group aged <24 months, who typically had the better prognosis. The most likely explanation for our observations is immune damage to bone marrow myeloid precursors, as described previously [23, 24], even though we cannot exclude unknown additional genetic factors that can cause a more severe clinical course. The lack of a statistically significant correlation between CFU and CD34+ cells may arise from the different distribution of the two kinds of analysis in infants and older children. While we have different control values for different pediatric ages for circulating CD34+ cells [20], our pediatric controls for CFU-GM are irrespective of age.

In conclusion, according to our data, the identification of defective myelopoiesis in a subgroup of children with chronic neutropenia might have clinical relevance, since these patients seem to have a higher incidence of infections and delayed remission. The evaluations of the absolute count of circulating CD34+ cells and the apoptotic rate are non-invasive and rapid [20]. This could easily allow a more stringent and careful follow up, especially for the group of patients aged <24 months, to identify subjects with a higher risk of delayed recovery.

## Supporting information

S1 Table Detailed description of patients with chronic neutropenia. (DOCX)
