# Supplementary material for: Reduction of CFU-GM and circulating hematopoietic progenitors in a subgroup of children with chronic neutropenia associated with severe infections and delayed recovery
Source: PLoS One. 2019 Mar 14;14(3):e0213782. doi: 10.1371/journal.pone.0213782 (PMC6417780; doi:10.1371/journal.pone.0213782)
Supplement: S1 Table — (DOCX) [file pone.0213782.s001.docx]

| patients | Age at diagnosis of neutropenia  (months) | ANC/μl  at diagnosis  of neutropenia | recovery | Follow-up  (months) | BM aspirate | Maturation  arrest | Dysplastic  features | CFU-GM | CFU-G | CD34+/ul | | AnnexV% | | Moderate-severe infections |
| --- | --- | --- | --- | --- | --- | --- | --- | --- | --- | --- | --- | --- | --- | --- |
| 1 | 5 | 470 | yes | 62 | yes |  |  | 2 | 11 | 2.9 | L | 16 | N |  |
| 2 | 48 | 513 | no | 180 | yes |  |  | 1 | 0 | 3.31 | N | 10.4 | H |  |
| 3 | 12 | 230 | no | 144 | Yes | yes |  | 15 | 3 | 2.2 | L | 51.4 | H | yes |
| 4 | 155 | 800 | no | 24 | - |  |  | - | - | 1.27 | L | 5.9 | N |  |
| 5 | 31 | 670 | yes | 120 | yes |  |  | 0 | 3 | 0.8 | L | 40.9 | H | yes |
| 6 | 168 | 290 | Yes | 43 | - |  |  | - | - | 1.7 | L | 34.0 | H |  |
| 7 | 11 | 367 | Yes | 66 | yes |  |  | 20 | 34 | 1.18 | L | 29.6 | H | yes |
| 8 | 9 | 150 | no | 40 | yes |  |  | 11 | 2 | 1.98 | L | 35.6 | H |  |
| 9 | 10 | 500 | Yes | 26 | - |  |  | - | - | 2.7 | L | 16.0 | N | yes |
| 10 | 18 | 440 | no | 44 | - |  |  | - | - | 1.36 | L | 3.8 | N |  |
| 11 | 9 | 235 | Yes | 8 | - |  |  | - | - | 12.4 | N | 7.1 | N |  |
| 12 | 8 | 440 | Yes | 29 | yes |  |  | 26 | 7 | 0.08 | L | 2.9 | N | yes |
| 13 | 6 | 380 | Yes | 70 | - |  |  | - | - | 2.4 | L | 18.3 | N | yes |
| 14 | 17 | 450 | Yes | 25 | yes |  |  | 2 | 2 | 0.8 | L | 35.0 | H |  |
| 15 | 14 | 930 | Yes | 14 | - |  |  | - | - | 2.65 | L | 17.5 | N |  |
| 16 | 8 | 250 | no | 90 | yes | yes | yes | 6 | 1 | 2.00 | L | 32.5 | H | yes |
| 17 | 27 | 350 | no | 84 | yes |  |  | 16 | 2 | - |  | - |  |  |
| 18 | 82 | 930 | Yes | 20 | - |  |  | - | - | 0.78 | L | 16.7 | N |  |
| 19 | 10 | 350 | no | 12 | - |  |  | - | - | 0.77 | L | 3.7 | N |  |
| 20 | 139 | 990 | Yes | 66 | yes |  |  | 153 | 83 | 1.1 | L | 42.1 | H |  |
| 21 | 13 | 60 | Yes | 9 | - |  |  | - | - | 6.4 | N | 6.3 | N |  |
| 22 | 48 | 480 | no | 148 | yes |  |  | 0 | 0 | 1.00 | L | 55.9 | H | yes |
| 23 | 192 | 700 | no | 13 | yes |  |  | 26 | 4 | 0.98 | L | 5.3 | N |  |
| 24 | 19 | 620 | Yes | 25 | - |  |  | - | - | 1.9 | L | 15.6 | N |  |
| 25 | 11 | 980 | Yes | 46 | - |  |  | - | - | 3.6 | N | 40.5 | H |  |
| 26 | 7 | 440 | Yes | 34 | yes |  |  | 58 | 53 | 10.0 | N | 23.2 | H |  |
| 27 | 11 | 408 | Yes | 24 | yes |  |  | 142 | 130 | 3.0 | L | 55.6 | H | yes |
| 28 | 6 | 570 | Yes | 9 | yes | yes |  | # | # | - | | - | | yes |
| 29 | 15 | 610 | Yes | 41 | yes |  |  | # | # | - | | - | |  |
| 30 | 3 | 490 | yes | 34 | yes |  |  | 57 | 62 | - | | - | |  |
| 31 | 12 | 440 | Yes | 9 | - |  |  | - | - | 5.4 | N | 5.8 | N |  |
| 32 | 12 | 600 | Yes | 38 | yes | yes |  | 48 | 56 | - | | - | | yes |
| 33 | 11 | 90 | Yes | 14 | - |  |  | - | - | 1.37 | L | 37.5 | H |  |
| 34 | 180 | 550 | no | 148 | yes |  |  | 2 | 2 | 0.7 | L | 80.0 | H | yes |
| 35 | 8 | 500 | Yes | 29 | yes |  |  | 62 | 70 | 7.0 | N | 69.7 | H | yes |
| 36 | 156 | 630 | Yes | 46 | - |  |  | - | - | 2.5 | N | 18.4 | N |  |
| 37 | 11 | 180 | no | 9 | yes |  |  | 30 | 7 |  | |  | | yes |
| 38 | 10 | 321 | no | 12 | - |  |  | - | - | 2.18 | L | 5.3 | N | yes |
| 39 | 51 | 430 | no | 23 | yes |  |  | 5 | 1 | 3.72 | N | 32.6 | H |  |
| 40 | 6 | 170 | Yes | 30 | yes |  |  | 7 | 0 | 4.76 | N | 6.9 | N |  |
| 41 | 7 | 920 | Yes | 8 | - |  |  | - | - | 4.22 | N | 42.7 | H |  |
| 42 | 12 | 195 | Yes | 43 | yes |  |  | 34 | 27 | 2.8 | N | 21.7 | N |  |
| 43 | 180 | 740 | Yes | 14 | yes |  |  | 1 | 1 | 0.5 | L | 84.5 | H |  |
| 44 | 4 | 506 | Yes | 17 | - |  |  | - | - | 3.9 | N | 53.2 | H |  |
| 45 | 72 | 330 | no | 60 | yes |  |  | 32 | 48 | 0.43 | L | 10.7 | N |  |
| 46 | 8 | 10 | Yes | 12 | yes |  |  | 35 | 27 |  | |  | |  |
| 47 | 8 | 100 | Yes | 11 | yes |  |  | 2 | 9 | 5.2 | N | 14.0 | N | yes |
| 48 | 76 | 140 | no | 49 | yes |  |  | # | # | 3.8 | N | 2.1 | N |  |
| 49 | 7 | 780 | no | 27 | yes |  |  | 15 | 7 | 1.35 | L | 7.1 | N |  |
| 50 | 18 | 180 | Yes | 16 | yes |  |  | 2 | 1 |  | |  | | yes |
| 51 | 12 | 10 | Yes | 24 | yes | yes |  | # | # |  | |  | | yes |
| 52 | 11 | 40 | Yes | 36 | - |  |  | - | - | 1.74 | L | 26.8 | H | yes |
| 53 | 18 | 600 | no | 52 | - |  |  | - | - | 1.72 | L | 11.9 | N |  |
| 54 | 7 | 10 | no | 15 | yes |  |  | 18 | 35 | 1.67 | L | 15.6 | N |  |
| 55 | 31 | 776 | Yes | 36 | - |  |  | - | - | 2.0 | L | 26.9 | H |  |
| 56 | 11 | 690 | Yes | 23 | - |  |  | - | - | 1.23 | L | 4.0 | N |  |
| 57 | 11 | 290 | Yes | 37 | yes |  |  | 29 | 11 | 8.1 | N | 49.4 | H | yes |
| 58 | 12 | 330 | Yes | 14 | - |  |  | - | - | 3.9 | L | 27.1 | H |  |
| 59 | 5 | 260 | Yes | 14 | yes | yes |  | 0 | 0 | - | | - | |  |
| 60 | 5 | 250 | Yes | 58 | yes |  |  | 5 | 28 | - | | - | | yes |
| 61 | 17 | 377 | Yes | 29 | - |  |  | - | - | 2.9 | N | 21.6 | N |  |
| 62 | 9 | 216 | Yes | 33 | - |  |  | - | - | 7.3 | N | 57.6 | H |  |
| 63 | 4 | 650 | Yes | 16 | - |  |  | - | - | 3.7 | L | 8.2 | N |  |
| 64 | 14 | 350 | Yes | 42 | - |  |  | - | - | 1.0 | L | 33.3 | H | yes |
| 65 | 7 | 190 | Yes | 7 | yes |  |  | 80 | 64 | 4.3 | N | 29.5 | H | yes |
| 66 | 9 | 100 | yes | 42 | yes |  |  | 28 | 20 | 3.31 | L | 44.4 | H |  |

# CFU not evaluable for technical reasons
